# Supplementary material for: Prevention of Gestational Diabetes Mellitus and Gestational Weight Gain Restriction in Overweight/Obese Pregnant Women: A Systematic Review and Network Meta-Analysis
Source: Nutrients. 2022 Jun 9;14(12):2383. doi: 10.3390/nu14122383 (PMC9231262; doi:10.3390/nu14122383)
Supplement: Supplementary file 1 [file nutrients-14-02383-s001.zip › Figure S1.pdf]

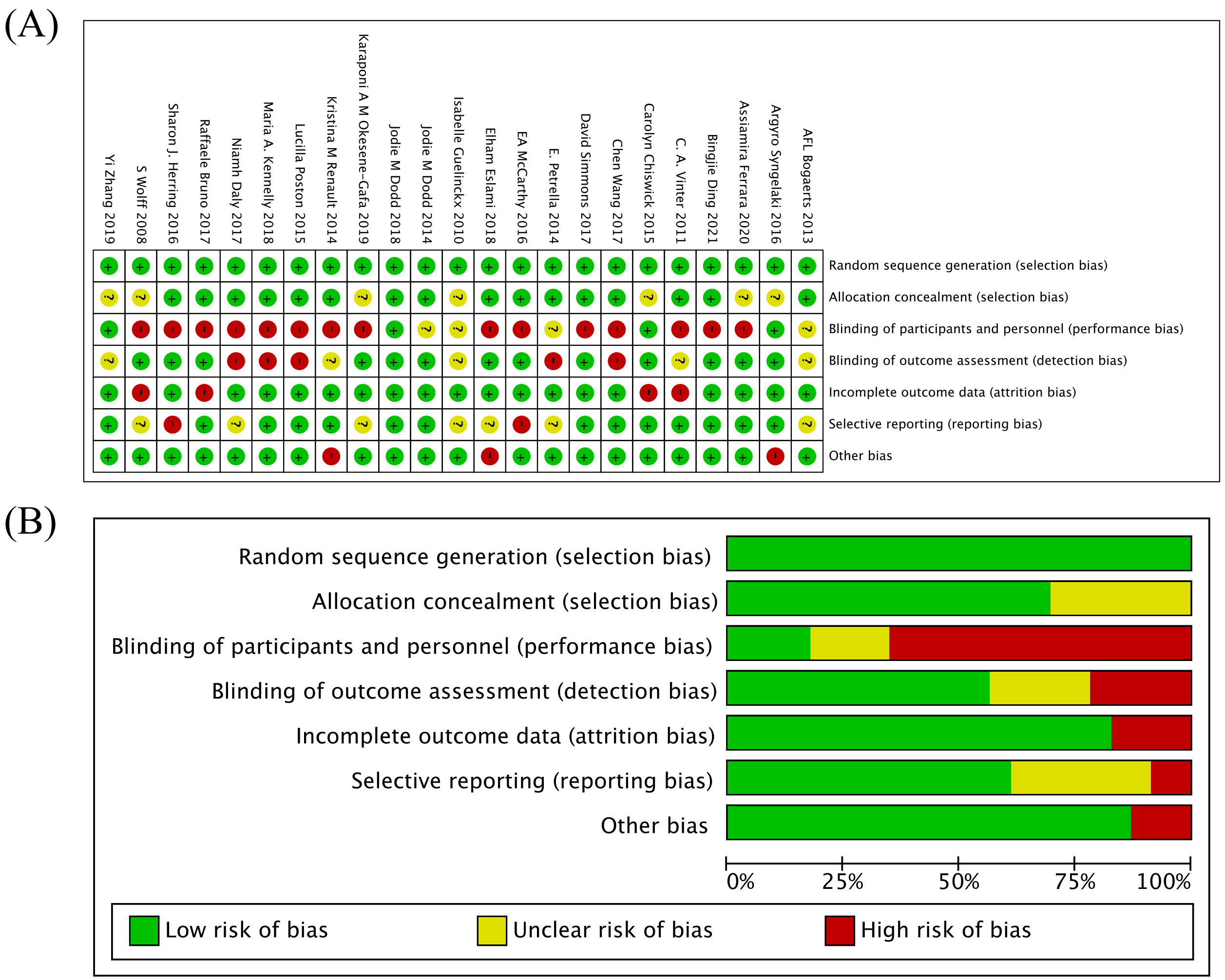

(A)
Yi Zhang 2019
S Wolff 2008
Sharon J. Herring 2016
Raffaele Bruno 2017
Niamh Daly 2017
Maria A. Kennelly 2018
Lucilla Poston 2015
Kristina M Renault 2014
Karaponi A M Okesene-Gafa 2019
Jodie M Dodd 2018
Jodie M Dodd 2014
Isabelle Guelinckx 2010
Elham Eslami 2018
EA McCarthy 2016
E. Petrella 2014
David Simmons 2017
Chen Wang 2017
Carolyn Chiswick 2015
C. A. Vinter 2011
Bingjie Ding 2021
Assiamira Ferrara 2020
Argyro Syngelaki 2016
AFL Bogaerts 2013
Random sequence generation (selection bias)
Allocation concealment (selection bias)
Blinding of participants and personnel (performance bias)
Blinding of outcome assessment (detection bias)
Incomplete outcome data (attrition bias)
Selective reporting (reporting bias)
Other bias
(B)
Random sequence generation (selection bias)
Allocation concealment (selection bias)
Blinding of participants and personnel (performance bias)
Blinding of outcome assessment (detection bias)
Incomplete outcome data (attrition bias)
Selective reporting (reporting bias)
Other bias
0%
25%
50%
75%
100%
Low risk of bias
Unclear risk of bias
High risk of bias
