# Supplementary material for: Prevention of Gestational Diabetes Mellitus and Gestational Weight Gain Restriction in Overweight/Obese Pregnant Women: A Systematic Review and Network Meta-Analysis
Source: Nutrients. 2022 Jun 9;14(12):2383. doi: 10.3390/nu14122383 (PMC9231262; doi:10.3390/nu14122383)
Supplement: Supplementary file 1 [file nutrients-14-02383-s001.zip › Figure S2.pdf]

(A)

Direct comparisons in the network

Network meta-analysis estimates	Diet vs PA	Diet vs Diet+PA	Diet vs Con	Diet+PA vs Con	PA vs Diet+PA	PA vs Con	Con vs Med
Mixed estimates							
Diet vs PA	12.0	5.6	38.4	0.1	5.5	38.5	·
Diet vs Diet+PA	6.1	27.9	21.4	4.2	23.3	17.2	·
Diet vs Con	10.1	5.1	65.5	1.0	4.1	14.2	·
Diet+PA vs Con	0.7	22.8	22.1	4.6	24.5	25.2	·
PA vs Diet+PA	5.8	22.7	16.8	4.4	29.1	21.2	·
PA vs Con	7.6	3.1	10.8	0.8	4.0	73.7	·
Con vs Med	·	·	·	·	·	·	100.0
Indirect estimates							
Diet vs Med	5.6	2.8	36.3	0.5	2.3	7.9	44.7
PA vs Med	4.1	1.7	5.8	0.5	2.1	39.8	46.0
Diet+PA vs Med	0.5	15.0	14.6	3.0	16.1	16.6	34.2
**Entire network**	4.9	11.6	22.7	2.1	12.1	24.5	22.2
**Included studies**	1	1	5	13	2	4	3

(B)

Direct comparisons in the network

Network meta-analysis estimates	Diet vs Con	Med vs Con	PA vs Con	Diet+PA vs Con
Mixed estimates				
Diet vs Con	100.0	·	·	·
Med vs Con	·	100.0	·	·
PA vs Con	·	·	100.0	·
Diet+PA vs Con	·	·	·	100.0
Indirect estimates				
Diet vs Med	50.0	50.0	·	·
Diet vs PA	50.0	·	50.0	·
Diet vs Diet+PA	50.0	·	·	50.0
Med vs PA	·	50.0	50.0	·
Med vs Diet+PA	·	50.0	·	50.0
PA vs Diet+PA	·	·	50.0	50.0
**Entire network**	25.0	25.0	25.0	25.0
**Included studies**	4	2	2	10
