# Supplementary material for: Prevention of Gestational Diabetes Mellitus and Gestational Weight Gain Restriction in Overweight/Obese Pregnant Women: A Systematic Review and Network Meta-Analysis
Source: Nutrients. 2022 Jun 9;14(12):2383. doi: 10.3390/nu14122383 (PMC9231262; doi:10.3390/nu14122383)
Supplement: Supplementary file 1 [file nutrients-14-02383-s001.zip › search strategy.pdf]

File S1. Search Strategy of This Network Meta-Analysis.

Pubmed:

#1: ((((((((Diabetes, Gestational[MeSH Terms]) OR (Diabetes, Pregnancy-Induced[Title/Abstract])) OR (Diabetes, Pregnancy Induced[Title/Abstract])) OR (Pregnancy-Induced Diabetes[Title/Abstract])) OR (Gestational Diabetes[Title/Abstract])) OR (Diabetes Mellitus, Gestational[Title/Abstract])) OR (Gestational Diabetes Mellitus[Title/Abstract])) OR (Diabetes, Gestational[Title/Abstract]) n=19993

#2: ((((((((((((((((((((((((((((((((((((((((((((((((((((((((((((((((((((((((Diet Therapy[MeSH Terms]) OR (Dietary Services[MeSH Terms])) OR (Diet[MeSH Terms])) OR (Exercise[MeSH Terms])) OR (Diet Therapy[Title/Abstract]) OR (Dietary Services[Title/Abstract])) OR (Diet[Title/Abstract])) OR (Exercise[Title/Abstract])) OR (Diet Therapies[Title/Abstract])) OR (Therapy, Diet[Title/Abstract])) OR (Diet Therapy, Restrictive[Title/Abstract])) OR (Restrictive Diet Therapies[Title/Abstract])) OR (Therapy, Restrictive Diet[Title/Abstract])) OR (Restrictive Diet Therapy[Title/Abstract])) OR (Restriction Diet Therapies[Title/Abstract])) OR (Diet Therapies, Restriction[Title/Abstract])) OR (Diet Therapy, Restriction[Title/Abstract])) OR (Therapy, Restriction Diet[Title/Abstract])) OR (Restriction Diet Therapy[Title/Abstract])) OR (Dietary Restriction[Title/Abstract])) OR (Dietary Restrictions[Title/Abstract])) OR (Restriction, Dietary[Title/Abstract])) OR (Dietary Modification[Title/Abstract])) OR (Dietary Modifications[Title/Abstract])) OR (Modification, Dietary[Title/Abstract])) OR (Diet Modification[Title/Abstract])) OR (Diet Modifications[Title/Abstract])) OR (Modification, Diet[Title/Abstract])) OR (nutritional management[Title/Abstract])) OR (dietary management[Title/Abstract])) OR (Services, Dietary[Title/Abstract])) OR (Dietary Service[Title/Abstract])) OR (Service, Dietary[Title/Abstract])) OR (Diets[Title/Abstract])) OR (dietary[Title/Abstract])) OR (diet consultation[Title/Abstract])) OR (dietary consultation[Title/Abstract])) OR (nutritional consultation[Title/Abstract])) OR (diet counseling[Title/Abstract])) OR (dietary counseling[Title/Abstract])) OR (nutritional counseling[Title/Abstract])) OR (diet intervention[Title/Abstract])) OR (diet interventions[Title/Abstract])) OR (dietary intervention[Title/Abstract])) OR (dietary interventions[Title/Abstract])) OR (Exercises[Title/Abstract])) OR (Physical

Activity[Title/Abstract])) OR (Activities, Physical[Title/Abstract])) OR (Activity, Physical[Title/Abstract])) OR (Physical Activities[Title/Abstract])) OR (Exercise, Physical[Title/Abstract])) OR (Exercises, Physical[Title/Abstract])) OR (Physical Exercise[Title/Abstract])) OR (Physical Exercises[Title/Abstract])) OR (Acute Exercise[Title/Abstract])) OR (Acute Exercises[Title/Abstract])) OR (Exercise, Acute[Title/Abstract])) OR (Exercises, Acute[Title/Abstract])) OR (Exercise, Isometric[Title/Abstract])) OR (Exercises, Isometric[Title/Abstract])) OR (Isometric Exercises[Title/Abstract])) OR (Isometric Exercise[Title/Abstract])) OR (Exercise, Aerobic[Title/Abstract])) OR (Aerobic Exercise[Title/Abstract])) OR (Aerobic Exercises[Title/Abstract])) OR (Exercises, Aerobic[Title/Abstract])) OR (Exercise Training[Title/Abstract])) OR (Exercise Trainings[Title/Abstract])) OR (Training, Exercise[Title/Abstract])) OR (Trainings, Exercise[Title/Abstract])) OR (lifestyle intervention[Title/Abstract])) OR (lifestyle interventions[Title/Abstract])) OR (lifestyle management[Title/Abstract])) OR (lifestyle managements[Title/Abstract]) n=1072177

#3: ((((((((((((((((((((((((((((((((((((((((((((((((((((((((((((((((((((((((((((((Hypoglycemic Agents[MeSH Terms])) OR (Glyburide[MeSH Terms])) OR (Metformin[MeSH Terms])) OR (Insulin[MeSH Terms])) OR (Hypoglycemic Agents[Title/Abstract])) OR (Glyburide[Title/Abstract])) OR (Metformin[Title/Abstract])) OR (Insulin[Title/Abstract])) OR (Agents, Hypoglycemic[Title/Abstract])) OR (Hypoglycemic Agent[Title/Abstract])) OR (Agent, Hypoglycemic[Title/Abstract])) OR (Antihyperglycemic Agent[Title/Abstract])) OR (Agent, Antihyperglycemic[Title/Abstract])) OR (Antihyperglycemics[Title/Abstract])) OR (Hypoglycemic[Title/Abstract])) OR (Hypoglycemic Drugs[Title/Abstract])) OR (Drugs, Hypoglycemic[Title/Abstract])) OR (Hypoglycemics[Title/Abstract])) OR (Antihyperglycemic Agents[Title/Abstract])) OR (Agents, Antihyperglycemic[Title/Abstract])) OR (Antihyperglycemic[Title/Abstract])) OR (Hypoglycemic Drug[Title/Abstract])) OR (Drug, Hypoglycemic[Title/Abstract])) OR (Antidiabetics[Title/Abstract])) OR (Antidiabetic Drug[Title/Abstract])) OR (Drug, Antidiabetic[Title/Abstract])) OR (Antidiabetic Drugs[Title/Abstract])) OR (Drugs, Antidiabetic[Title/Abstract])) OR (Antidiabetic Agents[Title/Abstract])) OR (Agents, Antidiabetic[Title/Abstract])) OR (Antidiabetic Agent[Title/Abstract])) OR (Agent,

Antidiabetic[Title/Abstract])) OR (Antidiabetic[Title/Abstract])) OR (Hypoglycemic Effect[Title/Abstract])) OR (Effect, Hypoglycemic[Title/Abstract])) OR (Hypoglycemic Effects[Title/Abstract])) OR (Effects, Hypoglycemic[Title/Abstract])) OR (Glybenclamide[Title/Abstract])) OR (Glibenclamide[Title/Abstract])) OR (Diabeta[Title/Abstract])) OR (Euglucon 5[Title/Abstract])) OR (Neogluconin[Title/Abstract])) OR (HB-419[Title/Abstract])) OR (HB 419[Title/Abstract])) OR (HB419[Title/Abstract])) OR (HB-420[Title/Abstract])) OR (HB 420[Title/Abstract])) OR (HB420[Title/Abstract])) OR (Maninil[Title/Abstract])) OR (Micronase[Title/Abstract])) OR (Daonil[Title/Abstract])) OR (Euglucon N[Title/Abstract])) OR (Dimethylbiguanidine[Title/Abstract])) OR (Dimethylguanylguanidine[Title/Abstract])) OR (Glucophage[Title/Abstract])) OR (Metformin Hydrochloride[Title/Abstract])) OR (Hydrochloride, Metformin[Title/Abstract])) OR (Metformin HCl[Title/Abstract])) OR (HCl, Metformin[Title/Abstract])) OR (Insulin, Regular[Title/Abstract])) OR (Regular Insulin[Title/Abstract])) OR (Soluble Insulin[Title/Abstract])) OR (Insulin, Soluble[Title/Abstract])) OR (Insulin A Chain[Title/Abstract])) OR (Sodium Insulin[Title/Abstract])) OR (Insulin, Sodium[Title/Abstract])) OR (Novolin[Title/Abstract])) OR (Iletin[Title/Abstract])) OR (Insulin B Chain[Title/Abstract])) OR (Chain, Insulin B[Title/Abstract]) n=469326

#4: (((((((Randomized Controlled Trial [Publication Type]) OR (Randomized Controlled Trials as Topic[MeSH Terms])) OR (Randomized Controlled Trial [Title/Abstract])) OR (Clinical Trials, Randomized[Title/Abstract])) OR (Trials, Randomized Clinical[Title/Abstract])) OR (Controlled Clinical Trials, Randomized[Title/Abstract])) OR (RCT[Title/Abstract])) OR (intervention study[Title/Abstract]) n=707576

#1 AND (#2 OR #3) AND #4 n=791

Embase:

#1: ‘pregnancy diabetes mellitus’/exp OR ‘gestational diabetes mellitus’:ab,ti OR ‘gestational diabetes’:ab,ti OR ‘diabetes mellitus, gestational’:ab,ti OR ‘diabetes, gestational’:ab,ti OR ‘diabetes, pregnancy induced’:ab,ti OR ‘pregnancy-induced diabetes’:ab,ti n=42000

#2: ‘diet therapy’/exp OR ‘physical activity’/exp OR ‘exercise’/exp OR ‘diet therapy’:ab,ti OR ‘diet therapies’:ab,ti OR ‘dietary therapy’:ab,ti OR ‘dietary therapies’:ab,ti OR ‘diet counseling’:ab,ti OR ‘dietary counseling’:ab,ti OR ‘diet consultation’:ab,ti OR ‘dietary consultation’:ab,ti OR ‘diet intervention’:ab,ti OR ‘dietary intervention’:ab,ti OR ‘diet interventions’:ab,ti OR ‘dietary interventions’:ab,ti OR ‘diet’:ab,ti OR ‘diets’:ab,ti OR ‘dietary’:ab,ti OR ‘physical activity’:ab,ti OR ‘physical activities’:ab,ti OR ‘exercise’:ab,ti OR ‘exercises’:ab,ti OR ‘exercise training’:ab,ti OR ‘exercise trainings’:ab,ti OR ‘aerobic exercise’:ab,ti OR ‘aerobic exercises’:ab,ti OR ‘physical training’:ab,ti OR ‘physical trainings’:ab,ti OR ‘lifestyle intervention’:ab,ti OR ‘lifestyle interventions’:ab,ti n=1732425

#3: ‘antidiabetic agent’/exp OR ‘glibenclamide’/exp OR ‘metformin’/exp OR ‘insulin’/exp OR ‘antidiabetic agent’:ab,ti OR ‘antidiabetic agents’:ab,ti OR ‘hypoglycemic agent’:ab,ti OR ‘hypoglycemic agents’:ab,ti OR ‘antiglycemic agent’:ab,ti OR ‘antiglycemic agents’:ab,ti OR ‘antidiabetic drug’:ab,ti OR ‘antidiabetic drugs’:ab,ti OR ‘hypoglycemic drug’:ab,ti OR ‘hypoglycemic drugs’:ab,ti OR ‘antiglycemic drug’:ab,ti OR ‘antiglycemic drugs’:ab,ti OR ‘glibenclamide’:ab,ti OR ‘glybenclamide’:ab,ti OR ‘glyburide’:ab,ti OR ‘metformin’:ab,ti OR ‘insulin’:ab,ti OR ‘insulins’:ab,ti n=737080

#4: ‘randomized controlled trial’/exp OR ‘randomized controlled trial’:ab,ti OR ‘randomized controlled study’:ab,ti OR ‘randomized clinical trial’:ab,ti OR ‘randomized clinical study’:ab,ti OR ‘RCT’:ab,ti OR ‘intervention study’:ab,ti n=726290

#1 AND (#2 OR #3) AND #4 n=1007

Cochrane Library:

#1: MeSH descriptor: [Diabetes, Gestational] explode all trees OR (Gestational Diabetes):ti,ab,kw OR (Diabetes, Pregnancy Induced):ti,ab,kw OR (Gestational Diabetes Mellitus):ti,ab,kw OR (Pregnancy-Induced Diabetes):ti,ab,kw OR (Diabetes Mellitus, Gestational):ti,ab,kw OR (Diabetes, Pregnancy-Induced):ti,ab,kw OR (Diabetes, Gestational):ti,ab,kw n=3355

#2: MeSH descriptor: [Diet Therapy] explode all trees OR MeSH descriptor: [Dietary Services] explode all trees OR MeSH descriptor: [Diet] explode all trees OR MeSH descriptor: [Exercise] explode all trees OR (Diet Therapy):ti,ab,kw OR (Dietary Services):ti,ab,kw OR (Diet):ti,ab,kw OR (Exercise):ti,ab,kw OR (Diet Therapies):ti,ab,kw OR (Therapy, Diet):ti,ab,kw OR (Diet Therapy, Restrictive):ti,ab,kw OR (Restrictive Diet Therapy):ti,ab,kw OR(Therapy, Restrictive Diet):ti,ab,kw OR (Restrictive Diet Therapies):ti,ab,kw OR (Restriction Diet Therapies):ti,ab,kw OR (Diet Therapies, Restriction):ti,ab,kw OR (Diet Therapy, Restriction):ti,ab,kw OR (Therapy, Restriction Diet):ti,ab,kw OR (Restriction Diet Therapy):ti,ab,kw OR (Dietary Restriction):ti,ab,kw OR(Dietary Restrictions):ti,ab,kw OR (Restriction, Dietary):ti,ab,kw OR (Dietary Modification):ti,ab,kw OR (Dietary Modifications):ti,ab,kw OR (Modification, Dietary):ti,ab,kw OR (Diet Modification):ti,ab,kw OR (Diet Modifications):ti,ab,kw OR (Modification, Diet):ti,ab,kw OR (nutritional management):ti,ab,kw OR (dietary management):ti,ab,kw OR(Services, Dietary):ti,ab,kw OR (Dietary Service):ti,ab,kw OR (Service, Dietary):ti,ab,kw OR (Diets):ti,ab,kw OR (dietary):ti,ab,kw OR (diet consultation):ti,ab,kw OR (dietary consultation):ti,ab,kw OR (nutritional consultation):ti,ab,kw OR (diet counseling):ti,ab,kw OR (dietary counseling):ti,ab,kw OR (nutritional counseling):ti,ab,kw OR (diet intervention):ti,ab,kw OR (diet interventions):ti,ab,kw OR (dietary intervention):ti,ab,kw OR (dietary interventions):ti,ab,kw OR (Exercises):ti,ab,kw OR (Physical Activity):ti,ab,kw OR (Physical Activities):ti,ab,kw OR (Activities, Physical):ti,ab,kw OR (Activity, Physical):ti,ab,kw OR (Exercise, Physical):ti,ab,kw OR (Exercises, Physical):ti,ab,kw OR (Physical Exercise):ti,ab,kw OR (Physical Exercises):ti,ab,kw OR (Acute Exercise):ti,ab,kw OR (Acute Exercises):ti,ab,kw OR (Exercise, Acute):ti,ab,kw OR (Exercises, Acute):ti,ab,kw OR

(Exercise, Isometric):ti,ab,kw OR (Exercises, Isometric):ti,ab,kw OR (Isometric Exercises):ti,ab,kw OR (Isometric Exercise):ti,ab,kw OR (Exercise, Aerobic):ti,ab,kw OR (Aerobic Exercise):ti,ab,kw OR (Aerobic Exercises):ti,ab,kw OR (Exercises, Aerobic):ti,ab,kw OR (Exercise Training):ti,ab,kw OR (Exercise Trainings):ti,ab,kw OR (Training, Exercise):ti,ab,kw OR (Trainings, Exercise):ti,ab,kw OR (lifestyle intervention):ti,ab,kw OR (lifestyle interventions):ti,ab,kw OR (lifestyle management):ti,ab,kw OR (lifestyle managements):ti,ab,kw n=181591

#3: MeSH descriptor: MeSH descriptor: [Hypoglycemic Agents] explode all trees OR MeSH descriptor: [Glyburide] explode all trees OR [Metformin] explode all trees OR MeSH descriptor: [Insulins] explode all trees OR (Hypoglycemic Agents):ti,ab,kw OR (Agents, Hypoglycemic):ti,ab,kw OR (Agent, Hypoglycemic):ti,ab,kw OR (Hypoglycemic Agent):ti,ab,kw OR (Antihyperglycemic Agent):ti,ab,kw OR (Agent, Antihyperglycemic):ti,ab,kw OR (Antihyperglycemics):ti,ab,kw OR (Hypoglycemic):ti,ab,kw OR (Hypoglycemic Drugs):ti,ab,kw OR (Drugs, Hypoglycemic):ti,ab,kw OR (Hypoglycemics):ti,ab,kw OR (Antihyperglycemic Agents):ti,ab,kw OR (Agents, Antihyperglycemic):ti,ab,kw OR (Antihyperglycemic):ti,ab,kw OR (Hypoglycemic Drug):ti,ab,kw OR (Drug, Hypoglycemic):ti,ab,kw OR (Antidiabetics):ti,ab,kw OR (Antidiabetic Drug):ti,ab,kw OR (Antidiabetic Drugs):ti,ab,kw OR (Drug, Antidiabetic):ti,ab,kw OR (Drugs, Antidiabetic):ti,ab,kw OR (Antidiabetic Agents):ti,ab,kw OR (Antidiabetic Agent):ti,ab,kw OR (Agents, Antidiabetic):ti,ab,kw OR (Agent, Antidiabetic):ti,ab,kw OR (Antidiabetic):ti,ab,kw OR (Hypoglycemic Effect):ti,ab,kw OR (Hypoglycemic Effects):ti,ab,kw OR (Effect, Hypoglycemic):ti,ab,kw OR (Effects, Hypoglycemic):ti,ab,kw OR (Glyburide):ti,ab,kw OR (Glybenclamide):ti,ab,kw OR (Glibenclamide):ti,ab,kw OR (Diabeta):ti,ab,kw OR (Euglucon 5):ti,ab,kw OR (Neogluconin):ti,ab,kw OR (HB-419):ti,ab,kw OR (HB 419):ti,ab,kw OR (HB419):ti,ab,kw OR (HB-420):ti,ab,kw OR (HB 420):ti,ab,kw OR (HB420):ti,ab,kw OR (Maninil):ti,ab,kw OR (Micronase):ti,ab,kw OR (Daonil):ti,ab,kw OR (Euglucon N):ti,ab,kw OR (Metformin):ti,ab,kw OR (Dimethylbiguanidine):ti,ab,kw OR (Dimethylguanylguanidine):ti,ab,kw OR (Glucophage):ti,ab,kw OR (Metformin Hydrochloride):ti,ab,kw OR (Hydrochloride, Metformin):ti,ab,kw OR (Metformin HCl):ti,ab,kw OR (HCl, Metformin):ti,ab,kw OR

(Insulins):ti,ab,kw OR (Insulin):ti,ab,kw (Insulin, Regular):ti,ab,kw OR (Regular Insulin):ti,ab,kw OR (Soluble Insulin):ti,ab,kw OR (Insulin, Soluble):ti,ab,kw OR (Insulin A Chain):ti,ab,kw OR (Sodium Insulin):ti,ab,kw OR (Insulin, Sodium):ti,ab,kw OR (Novolin):ti,ab,kw OR (Iletin):ti,ab,kw OR (Insulin B Chain):ti,ab,kw OR (Chain, Insulin B):ti,ab,kw n=67934

#4: MeSH descriptor: [Randomized Controlled Trial] explode all trees OR (Randomized Controlled Trial):ti,ab,kw OR (Clinical Trials, Randomized):ti,ab,kw OR (Trials, Randomized Clinical):ti,ab,kw OR (Controlled Clinical Trials, Randomized):ti,ab,kw OR (RCT):ti,ab,kw OR (intervention study):ti,ab,kw n=754453

#5: #1 AND (#2 OR #3) AND #4 n=1499

Web of Science:

#1: TS= (Diabetes, Gestational) OR TS=(Diabetes, Pregnancy-Induced) OR TS=(Diabetes, Pregnancy Induced) OR TS=(Pregnancy-Induced Diabetes) OR TS=(Gestational Diabetes) OR TS=(Diabetes Mellitus, Gestational) OR TS=(Gestational Diabetes Mellitus) OR TS=(Diabetes, Gestational) n=39608

#2: TS= (Diet Therapy) OR TS=(Diet Therapies) OR TS=(Diet) OR TS=(Diets) OR TS=(Dietary) OR TS=(diet consultation) OR TS=(dietary consultation) OR TS=(diet counseling) OR TS=(dietary counseling) OR TS=(diet intervention) OR TS=(diet interventions) OR TS=(dietary intervention) OR TS=(dietary interventions) OR TS=(Exercise) OR TS=(Exercises) OR TS=(Physical Activity) OR TS=(Physical Activities) OR TS=(Aerobic Exercise) OR TS=(Aerobic Exercises) OR TS=(physical training) OR TS=(physical trainings) OR TS=(lifestyle intervention) OR TS=(lifestyle interventions) n=3342783

#3: TS= (Hypoglycemic Agent) OR TS= (Hypoglycemic Agents) OR TS=(Antihyperglycemic Agent) OR TS=(Antihyperglycemic Agents) OR TS=(Antidiabetic Agent) OR TS=(Antidiabetic Agents) OR TS= (Hypoglycemic Drug) OR TS= (Hypoglycemic Drugs) OR TS=(Antidiabetic Drug) OR TS=(Antidiabetic Drugs) OR TS=(Glyburide) OR TS=(Glybenclamide) OR TS=(Glibenclamide) OR TS=(Metformin) OR TS=(Dimethylbiguanidine) OR TS=(Dimethylguanylguanidine) OR TS=(Glucophage) OR TS=(Insulin) OR TS=(Insulins) n=970554

#4: TS= (Randomized Controlled Trial) OR TS= (Clinical Trials, Randomized) OR TS=(Trials, Randomized Clinical) OR TS=(Controlled Clinical Trials, Randomized) OR TS=(RCT) OR TS=(intervention study) n=1679638

#5: #1 AND (#2 OR #3) AND #4 n=2698
