# Supplementary figures and images for: Prevention of Gestational Diabetes Mellitus and Gestational Weight Gain Restriction in Overweight/Obese Pregnant Women: A Systematic Review and Network Meta-Analysis
Source: Nutrients. 2022 Jun 9;14(12):2383. doi: 10.3390/nu14122383 (PMC9231262; doi:10.3390/nu14122383)

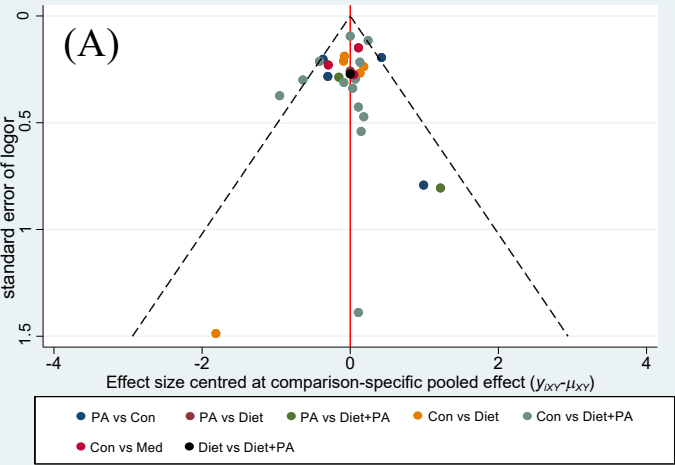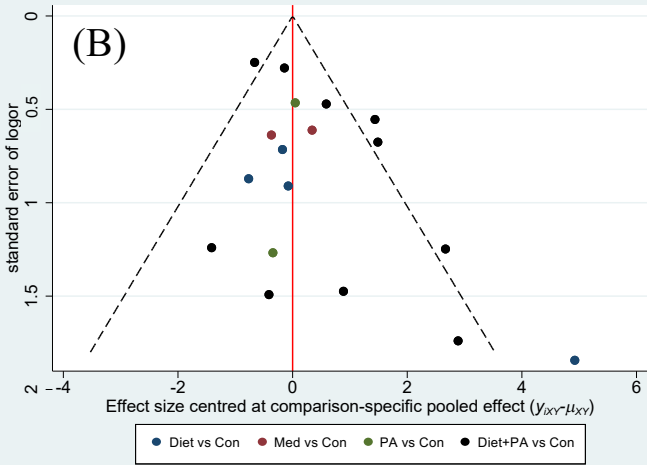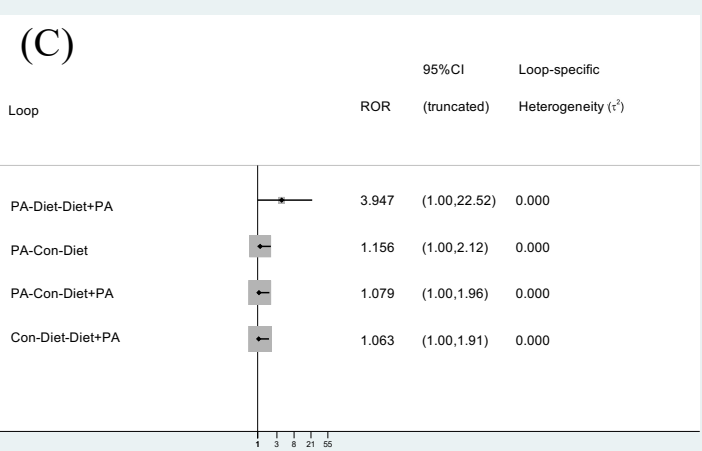

Supplement: Supplementary file 1 [file nutrients-14-02383-s001.zip › Figure S3-new.pdf]
